# Supplementary material for: Clinical characteristics, anti-SARS-CoV-2 IgG titers, and inflammatory markers in individuals with post-COVID-19 condition in Kenya: a cross-sectional study
Source: PeerJ. 2024 Jul 22;12:e17723. doi: 10.7717/peerj.17723 (PMC11271650; doi:10.7717/peerj.17723)
Supplement: Supplemental Information 3 [file peerj-12-17723-s003.docx]

|  |
| --- |
| ***SARS-CoV-2 Sero-surveillance study at KU Health Unit*** |
| **Socio-Demographics** |
| **Instructions: Please select the best answer of your choice.** |
| 1.How old are you in years ?________ |
| 2. What is your gender?  **O** Male  **O** Female |
| 3. What is your highest level of education?  **O** Primary school  **O** Secondary school  **O** College and above  **O** No formal education |
| 4. What is your current occupation  **O** Teaching staff  **O** Non-teaching staff  **O** Student  **O** Health unit staff  **O** Student  **O** Other (Specify)____________________ |
| 5. Have you been sick with coronavirus (Covid-19)?  **O** Yes, confirmed  **O** Yes, but not yet confirmed  **O** No |
| 6.If yes, when was the last time you were confirmed sick with Covid-19?________  7. If you answered Yes to Question 5, how ill were you with Covid 19?  O I did not have any symptoms  O I had symptoms but recovered without medication  O I had symptoms and was treated as an outpatient in hospital  O I had symptoms and was admitted to hospital in the general wards  O I had symptoms and was admitted to hospital in the ICU/HDU  8.   \| When you had Covid-19 which symptoms did you have? Please select all that apply. \| \| \| \| --- \| --- \| --- \| \|  \| Yes \| No \| \| Fever \| O \| O \| \| Cough \| O \| O \| \| Shortness of breath \| O \| O \| \| Sore throat \| O \| O \| \| Runny or stuffy nose \| O \| O \| \| Muscle or body aches \| O \| O \| \| Headaches \| O \| O \| \| Fatigue (tiredness) \| O \| O \| \| Diarrhea \| O \| O \| \| Loss of taste and smell \| O \| O \|   9. How long did you take to recover from COVID-19 symptoms  Less than 2 weeks  2 weeks – 4 weeks  1 month to 3 months  3 months to a year  More than a year  I still have symptoms  10. If you still have symptoms, please tick which of them still persist   \|  \| Yes \| No \| \| --- \| --- \| --- \| \| Fever \| O \| O \| \| Cough \| O \| O \| \| Shortness of breath \| O \| O \| \| Sore throat \| O \| O \| \| Runny or stuffy nose \| O \| O \| \| Muscle or body aches \| O \| O \| \| Headaches \| O \| O \| \| Fatigue (tiredness) \| O \| O \| \| Diarrhea \| O \| O \| \| Loss of taste and smell \| O \| O \|   11. Did you develop any symptoms that were not there at your initial diagnosis later on in your illness?  Yes  No  12. If you answered Yes to question 11, please list the symptoms you experienced (tick all that apply)  Extreme Tiredness  Shortness of breath  Loss of smell or taste  Muscle aches and pains  Heart palpitations  Dizziness  Pins and needles  Joint pain  Depression and anxiety  Earache and tinnitus  Loss of appetite and nauseas  Skin rash  Problems with memory or concentration’ |
| 13. Have you been vaccinated against coronavirus (Covid-19)?  O Yes  O No |
| 14. If yes, what vaccine did you receive?  O MODERNA  O ASTRAZENECA  O PFIZER  O JOHNSON&JOHNSON  O SINOPHARM  O DON’T KNOW  15. If yes, have you received a booster dose?_______  16. If yes, what booster dose did you receive?  O MODERNA  O ASTRAZENECA  O PFIZER  O JOHNSON&JOHNSON  O SINOPHARM  O DON’T KNOW  17.When last did you receive your vaccine?_______  18.Have you been infected with Covid-19 following vaccination?_______  19. If you answered Yes to Question 18, how ill were you with Covid 19 after vaccination?  O I did not have any symptoms  O I had symptoms but recovered without medication  O I had symptoms and was treated as an outpatient in hospital  O I had symptoms and was admitted to hospital in the general wards  O I had symptoms and was admitted to hospital in the ICU/HDU  20 When were you last diagnosed with Covid-19?____________ |
| 21. If you have not been vaccinated against Covid-19, please indicate why?______________________________________________________ |
